# N_2_O production, a widespread trait in fungi

**DOI:** 10.1038/srep09697

**Published:** 2015-04-20

**Authors:** Koki Maeda, Aymé Spor, Véronique Edel-Hermann, Cécile Heraud, Marie-Christine Breuil, Florian Bizouard, Sakae Toyoda, Naohiro Yoshida, Christian Steinberg, Laurent Philippot

**Affiliations:** 1 NARO, Hokkaido Agricultural Research Center, Dairy Research Division, 1 Hitsujigaoka, Sapporo 062-8555, Japan; 2 INRA, UMR 1347 Agroécologie, 17 rue Sully, 21065 Dijon Cedex, France; 3 Department of Environmental Science and Technology, Tokyo Institute of Technology, 4259 Nagatsuta, Midori-ku, Yokohama 226-8502, Japan; 4 Department of Environmental Chemistry and Engineering, Tokyo Institute of Technology, 4259 Nagatsuta, Midori-ku, Yokohama 226-8502, Japan; 5 Earth-Life Science Institute, Tokyo Institute of Technology, 2-12-1 Ookayama, Meguro-ku, Tokyo 152-8550, Japan

## Abstract

N_2_O is a powerful greenhouse gas contributing both to global warming and ozone depletion. While fungi have been identified as a putative source of N_2_O, little is known about their production of this greenhouse gas. Here we investigated the N_2_O-producing ability of a collection of 207 fungal isolates. Seventy strains producing N_2_O in pure culture were identified. They were mostly species from the order *Hypocreales* order—particularly *Fusarium oxysporum* and *Trichoderma* spp.—and to a lesser extent species from the orders *Eurotiales, Sordariales, and Chaetosphaeriales.* The N_2_O ^15^N site preference (SP) values of the fungal strains ranged from 15.8‰ to 36.7‰, and we observed a significant taxa effect, with *Penicillium* strains displaying lower SP values than the other fungal genera. Inoculation of 15 N_2_O-producing strains into pre-sterilized arable, forest and grassland soils confirmed the ability of the strains to produce N_2_O in soil with a significant strain-by-soil effect. The copper-containing nitrite reductase gene (*nirK*) was amplified from 45 N_2_O-producing strains, and its genetic variability showed a strong congruence with the *ITS* phylogeny, indicating vertical inheritance of this trait. Taken together, this comprehensive set of findings should enhance our knowledge of fungi as a source of N_2_O in the environment.

Terrestrial ecosystems are a major source of nitrous oxide (N_2_O)^1^,^2^, a so-called greenhouse gas also commonly known as laughing gas. Although it has received much less attention than CO_2_, the 100 year global warming potential of N_2_O is 298 times greater than that of CO_2_ due to the much longer half life of N_2_O^3^. There is also growing concern over nitrous oxide concentrations because, following the reduction of chlorine- and bromine-containing halocarbons by the Montreal Protocol, N_2_O has become the main ozone-depleting substance emitted to the stratosphere^4^.

Nitrous oxide emissions are mostly due to two microbial processes: nitrification and denitrification. Nitrous oxide is a by-product of the first step of nitrification, the oxidation of ammonia to nitrite^5^. In contrast, N_2_O is either an intermediate or the end product of the denitrification cascade, which consists in the reduction of nitrate or nitrite into nitric oxide, nitrous oxide and dinitrogen^6^. Sixty-two percent of the total global N_2_O emissions are from natural and agricultural soils (6 and 4.2 Tg N yr^−1^, respectively^7^), and denitrification is traditionally considered as the main source of these emissions^8^.

It is well known that denitrification is widespread among prokaryotes—indeed, the ability to denitrify has been observed in more than 60 bacterial and archaeal genera^9^. Moreover, eukaryotes such as fungi in soils^10^ or foraminifers in aquatic environments^11^,^12^ are also capable of denitrification. Characterization of the fungal denitrification ability in *Fusarium oxysporum* and *Cylindrocarpon tonkinense* has shown that this reductive process was performed via a copper-containing nitrite reductase (NirK) and cytochrome P450 nitric oxide reductase^10^. However, no nitrous oxide reductase has been identified in fungi and N_2_O is the end product of denitrification in the few characterized fungal strains^13^,^14^. By using fungal or bacterial inhibitors to distinguish the microbial origin of N_2_O, previous studies have reported that fungi could contribute up to 18% of potential denitrification^15^ and be significant N_2_O producers in some terrestrial systems^16^,^17^. Despite the importance of fungi in several soil functions, such as organic matter decomposition^18^ and primary production through symbiotic or pathogenic relationships with plants^19^, the production of N_2_O by fungi has only been studied in a limited number of strains^14^,^20^. To what extent this trait is conserved amongst fungi remains unknown, but understanding the microbial sources of this greenhouse gas will be crucial for selecting mitigation strategies. Here, we screened a collection of 207 fungal strains belonging to 9 classes and 23 orders to determine the prevalence of the N_2_O-producing capacity among fungi. We further characterized the initial and end-products of denitrification of the N_2_O production-positive strains in pure culture and determined their N_2_O isotopic signature. Positive fungal strains were also inoculated into pre-sterilized arable, forest and grassland soils in order to verify their ability to produce this greenhouse gas in soil. Finally, we studied the phylogeny of the *nirK* gene, which encodes the copper-containing nitrite reductase using newly developed primers, and investigated the relationships between the nuclear ribosomal internal transcribed spacer (ITS) region and *nirK* phylogeny, N_2_O production rates and N_2_O isotopic signatures.

## Results and Discussion

**Figure 1:**
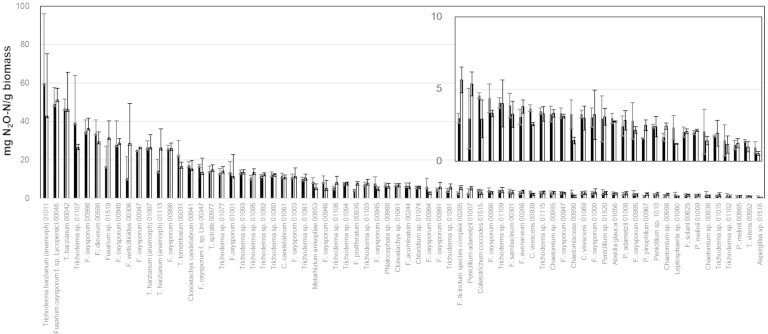
N_2_O production by the positive fungi strains with (white) and without (black) 10% C_2_H_2_ in the headspace. Error bars indicate the standard deviation (n = 3). Both strain names and MIAE numbers are indicated.

To assess how the N_2_O producing ability is distributed within fungi, 207 fungal strains comprising 23 orders and 54 genera were screened by incubating the strains in liquid culture under conditions that were previously reported to favour fungal denitrification^21^. The strains were selected to cover the largest possible fungal diversity within the Microorganisms of Interest for Agriculture and Environment collection (MIAE) (INRA, Dijon, France), which is dedicated to soil microbial diversity. At the end of the incubation, differences in the pH of the media were observed between strains. Since N_2_O can also be produced by chemical denitrification at low pH^22^, abiotic N_2_O production from nitrite was evaluated in sterile media with a pH gradient, and strains were scored positive when the N_2_O concentrations in the headspace were higher than those in the sterile flasks at the same pH (Fig. S1). When nitrite was used as an electron acceptor, more than a third of the strains were capable of producing N_2_O, with activities ranging from 0.5 ± 0.1 to 60.0 ± 36.0 mg N_2_O-N g^−1^ dry fungal biomass (Fig. 1). The N_2_O-producing activities were much lower when nitrate was used as an electron acceptor (<0.1 mg N_2_O-N g^−1^ fungal biomass; F = 108.55, *P* < 0.0001), supporting previous studies showing that nitrite rather than nitrate is preferable for fungal denitrification^23^. No difference was observed when incubating the positive strains with and without acetylene, indicating that the fungi does not reduce N_2_O, which was also in accordance with previous studies^24^. Accordingly, amplification of the *nosZ* gene using various primer sets^25^,^26^ was not successful (data not shown). The high proportion of N_2_O-producing fungal strains observed in our study contrasts with previous studies in which only 1% to 10% of examined bacteria were capable of denitrification based either on culture-based, direct-molecular approaches or genome analyses^27^,^28^,^29^. However, the maximum percentage of nitrogen recovered as N_2_O from nitrite in our work was about 38%, and most of the fungal strains reduced between 3% and 10% of the nitrite, while denitrifying bacteria are capable of reducing at least 80% of soluble nitrogen into gas^30^. Nonetheless, lower percentages were also reported for denitrifying bacteria such as *Bacillus* species, with ranges between 3.5% and 13.2% of the nitrate reduced to gaseous nitrogen after 48 h of growth^31^. Although we cannot rule out that growing the strains in different media or conditions may have resulted in different rates of nitrogen reduction, our incubation experiment using the standard media and conditions showed that N_2_O production is common within the fungal kingdom.

Fifty out of the 70 positive strains belonged to the *Hypocreales* order, and *Fusarium* and *Trichoderma* were the main *Hypocreales* genera observed. Interestingly, many of the *Fusarium* strains identified as N_2_O-producers were *Fusarium oxysporum*. Even within this species, the production rate was highly variable, ranging from 2.8 to 34.7 mg N_2_O-N g^−1^ dry fungal biomass. This species was reported to be one of the dominant fungal taxa in several studies^32^, and accounted for up to 43% of the ITS pyrosequencing dataset retrieved from Mediterranean soils^33^. *F. oxysporum* includes non-pathogenic and pathogenic strains, with the latter causing disease to a broad range of host plants, but no plant-based bioassay has been conducted on the tested strains to discriminate pathogenic and non-pathogenic *F. oxysporum*. In any case, the high number of N_2_O-producing *F. oxysporum* individuals suggests this species is involved in greenhouse gas emissions, and therefore are potentially detrimental in terms of both primary production and climate regulation. In both *Trichderma* and *Fusarium* species capable of living in plant tissue, respiration of nitrogen oxides is likely due to the adaptation to hypoxic conditions. Indeed, the oxygen concentration near or within plant tissue is low (<1%)^34^, and several studies have reported that it is critical to the fungal infection of plants that the infecting fungus possess strategies to overcome hypoxia^35^. The other positive strains identified in this work belong to the *Eurotiales* (8), *Sordariales* (5), *Chaetosphaeriales* (3), *Mucorales* (1), *Pleosporales* (1), *Glomerellales* (1) and *Ophiostomatales* orders (1). *Sordariales* has also been reported as a dominant order in agricultural soil, while the order *Pleosporales* is frequently detected in that environment^32^. Collectively, our results indicate that N_2_O production is a common trait in fungal taxa that are frequently abundant in soils, although this feature seems to be more a strain-specific than a species-specific trait. It also underlines the importance of quantifying the fungal contribution to terrestrial N_2_O emissions.

Analysis of the N_2_O isotopomer ratios (relative abundance of ^14^N^15^N^16^O or ^15^N^14^N^16^O to that of ^14^N^14^N^16^O) has been proposed as a powerful method to obtain more information on the sources of this greenhouse gas^36^. However, the site preference (SP) values due to fungal N_2_O production have only been determined in 2 strains (*F. oxysporum* and *C. tonkinense*)^37^, and a more comprehensive analysis of the variability of the fungal 15N site preference is required for a robust distinction between the bacterial and fungal contributions to N_2_O emissions. The SP values of the 67 fungal strains tested herein varied from 15.8–36.7‰ with an average value of 30.0 ± 4.8‰. Our results are partly consistent with the previous study reporting a positive SP of 36.9–37.1‰^37^. However, we found a larger range of variation, with SP values as low as 15.8 ± 2.6‰ for *Penicillium melinii* and as high as 36.7 ± 2.2‰ for *F. sambucinum*. These strains with low SP values often had more acidic conditions at the end of the incubation (pH 3–5; Fig. S1). Therefore, we cannot rule out that these lower SP values resulted from the higher contribution of abiotic N_2_O production under more acidic conditions^38^. Interestingly, a significant taxa effect (*P* < 0.01 by Student's *t*-test) with lower SP values was observed for the *Penicillium* strains compared to the other fungal genera. We thus confirmed that the N_2_O isotopic signature can be used to determine which organisms, *i.e.*, fungi or bacteria, are producing N_2_O by denitrification because of their distinct SP (about 0–10‰ for bacterial denitrification^39^). However, it will be difficult to distinguish N_2_O emissions from nitrification and fungal denitrification in the environment, since SP values for the N_2_O produced by both bacteria and archaea during ammonia oxidization ranged between 13.1 and 30.2‰^40^,^41^. Since fungi are microaerophilic denitrifiers, our results suggest that a stable isotopic approach alone is not enough to decipher whether nitrification or fungal denitrification is contributing to N_2_O emissions in environments where fungi are abundant.

**Figure 2:**
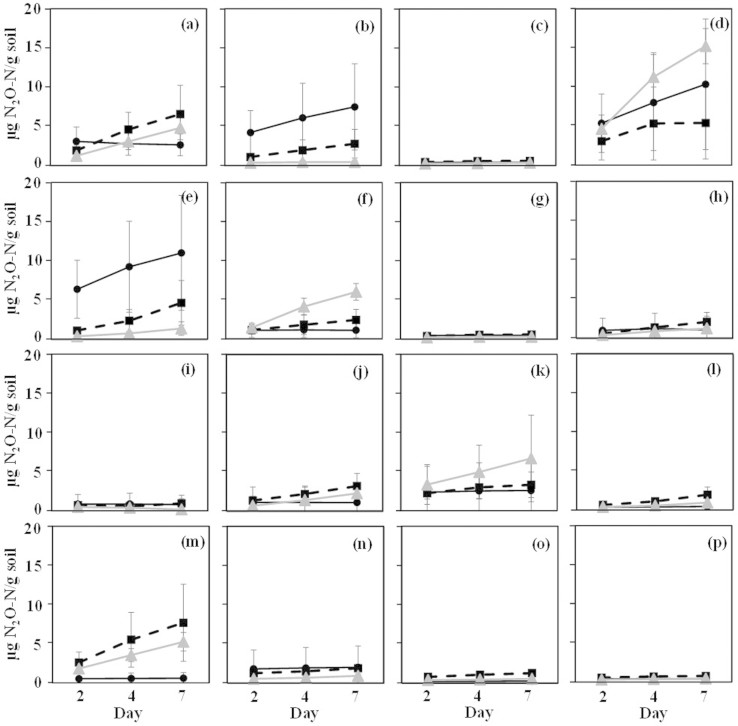
N_2_O production by 15 selected fungi strains inoculated in 3 different sterile soils. NO_2_^−^ was used as the electron acceptor. The strains were incubated for 7 days, and headspace N_2_O concentration were measured 3 times (2, 4 and 7 days after NO_2_^−^ addition). Code for the soils: Black circle: Sweden (Slogaryd), forest soil; grey triangle: France, arable soil; black rectangle: The Netherlands, grassland soil. Code for the fungi: a: *Trichoderma harzianum* (MIAE00042); b: *Fusarium verticillioides* (MIAE00306); c: *Penicillium adametzii* (MIAE01008); d: *F. oxysporum* f. sp. *lini* (MIAE00347); e: *F. dimerum* (MIAE00598); f: *Metarhizium anisopliae* (MIAE00953); g: *Chaetomium* sp. (MIAE00985); h: *T. harzianum* (anamorph) (MIAE01011); i: *Leptosphaeria* sp. (MIAE01060); j: *T. tomentosum* (MIAE00031); k: *Fusarium* sp. (MIAE01519); l: *Clonostachys candelabrum* (MIAE00941); m: *Phialocephala* sp. (MIAE00968); n: *Colletotrichum coccodes* (MIAE01515); o: *Aspergillus* sp. (MIAE01518);); p: No fungi control. The error bar represents the standard deviation (n = 3).

To further confirm that fungi could actually produce N_2_O in soil and not only in liquid culture, 15 of the positive strains were selected based on their high activity in pure culture and/or their taxonomic affiliation for inoculation into an arable, a grassland and a forest pre-sterilized soils. After allowing soil colonization by the inoculated fungal strains for a month, nitrite was added to induce denitrification and the concentration of N_2_O in the headspace was measured after 2, 4 and 7 days. Seven strains belonging to the *Trichoderma, Fusarium, Penicillium* and *Phialocephala* genera produced N_2_O in at least one of the soils, up to a maximum amount of 82.1 ng N_2_O-N g^−1^ h^−1^ (Fig. 2), which was significantly higher (F = 15590, *P* < 0.0001) than the amount produced by chemical denitrification in the non-inoculated sterile soils. This is comparable to previous studies focusing on fungal contribution^15^,^16^,^17^,^42^ or net N_2_O production from soil^43^,^44^,^45^. In our study, the highest amount of N_2_O produced was observed in the forest soil for *Fusarium verticillioides* and *F. dimerium*, while the grassland and the arable soils were the soils with the highest emissions for *Trichoderma harzianum* and *Phialocephala* spp. on one hand, and *F. oxysporum* f. sp. *lini*, *Metarhizium anisopliae* on the other. These significant strain-by-soil effects (F = 4.69, *P* < 0.0001) likely reflect differences in fungal nutrient requirements and/or preferences for different soil physico-chemical characteristics. Indeed, previous studies demonstrated that several soil physico-chemical parameters, such as extractable P concentrations, C/N ratio^46^, pH, sand content or litter cover^47^, can affect the soil fungal communities or fungal biomass^48^. The fungal N_2_O-producing capacity observed in our study could also be influenced by biotic interactions occurring in the natural ecosystems between fungi or with bacteria and other organisms.

**Figure 3:**
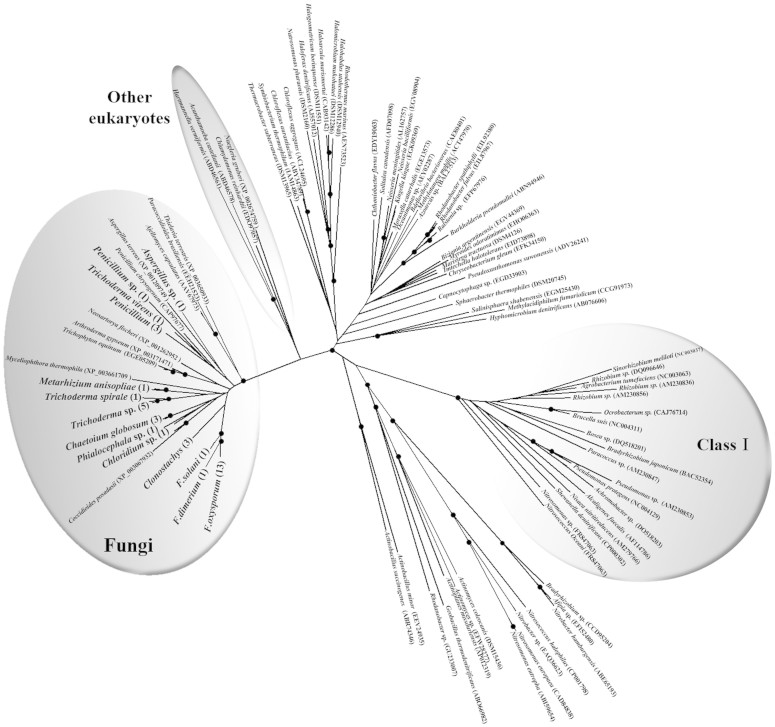
Neighbour-joining phylogenetic tree of *nirK* amino acid sequences constructed by Clustal W with 1000 bootstrap samplings. Strain names in bold indicate the sequences obtained in this study. The numbers in parentheses indicate the number of the strains. Bootstrap values greater than 75% are indicated as black circles.

Both copper-containing nitrite reductase (encoded by the *nirK* gene) and P450nor (nitric oxide reductase) are key enzymes involved in fungal denitrification. However, P450nor belongs to a superfamily of proteins that are widely distributed among fungi and known to be involved in a wide variety of physiological reactions^10^, which prevents the use of the corresponding genes as molecular markers to target denitrifying fungi. To date, several primers targeting the *nirK* denitrification gene have been described in the literature, but none of them was designed to amplify denitrifying eukaryotes^25^,^49^. Despite the low number of fungal *nirK* sequences available in the databases (less than 30) and the high diversity of the tested fungal strains, the amplification of the fungal *nirK* denitrification gene was successful in 45 out of 70 strains using our newly designed primer set EunirK-F1 and EunirK-R2. This supports our findings that these fungal strains are capable of denitrification and that N_2_O was not produced by other processes. Notably, when used to amplify DNA extracted from soil, our primer set also amplified the bacterial *nirK* in part due to the lower proportion of fungi in soil compared to bacteria (data not shown). A phylogenetic tree was constructed using these fungi *nirK* sequences and bacterial *nirK* sequences from available databases (Fig. 3). Our fungal *nirK* sequences clustered with the other fungal *nirK* homologues retrieved from the database, and were distinct from the bacterial *nirK* sequences. The phylogeny also shows that fungal *nirK* sequences are closer to other eukaryotic sequences (amoeba, protozoa or green alga) than to bacterial ones, indicating that fungal *nirK* sequences branched from bacterial *nirK* sequences at a very early stage of their evolutional history, as suggested by Kim et al.^50^. In addition, we also found a strong congruence between the *ITS* and the *nirK* phylogenies (Fig. S2), indicating a vertical inheritance of *nirK* genes. Interestingly, we found no correlation between the genetic distance of the *nirK* genes and the N_2_O production rates. Similarly, weak or no relationships were observed between the bacterial genotypes and denitrification phenotypes in previous studies^31^,^51^. Phenotypic convergence within similar ecological niches of distantly related organisms can lead to such a discrepancy between genetic and phenotypic distances. In depth investigation of the ecology of denitrifying fungi would undoubtedly help clarify which environmental factors lead to a convergent denitrification phenotype.

In conclusion, the analysis of a vast collection of fungi showed that N_2_O production is a common and widespread trait in fungi. Nitrite instead of nitrate was the preferred substrate, while N_2_O was always the end-product of denitrification. We showed that the range of variation of the N_2_O isotopic signature was taxa-dependent and larger than previously reported, with values as low as 15%. Inoculation of 15 strains into previously sterilized arable, forest and grassland soils demonstrated the ability of fungi to contribute to soil N_2_O emissions with fluxes potentially as high as those reported in natural soils. Further studies are clearly warranted to elucidate the significance of denitrification in fungi and its consequences for N_2_O emissions.

## Methods

### Fungal strains and in vitro incubation experiments

The fungal strains tested in this study were previously isolated from agricultural soils or plant roots. The details of the isolating procedures have been described previously^52^. The strains were purified by single-spore isolation and preserved in the MIAE collection (INRA, Dijon, France). Fungal strains were first cultured under an aerobic condition in a 147 ml plasma flask containing 50 ml of liquid malt medium (pH 7.5). The flasks were incubated with a rotator shaker at 25°C, 120 rpm. After 3 days to 2 weeks depending on their growth, 50 ml of liquid malt medium with 10 mM NaNO_2_ (pH 7.5) was added to the plasma flasks, which were capped with a butyl rubber stopper. The headspace gas was then replaced by pure N_2_ gas and 1 ml of pure O_2_ to obtain microaerobic conditions. The flasks were incubated again at 25°C, 120 rpm for one additional week. The incubations were performed in triplicate flasks for each fungal strain. After the second incubation round, 0.5 ml of headspace gas was sampled and N_2_O concentrations were determined by gas chromatography. Three previously characterized strains^24^,^53^,^54^, *F. oxysporum* MT811 (JCM11502), *Cylindrocarpon lichenicola* (NBRC30561) and *Aspergillus oryzae* RIB40 (NBRC100959), were used as positive controls. At the end of the incubation, mycelia were collected and dried for 24 h at 105°C for fungal biomass determination, and the pH of the medium was determined by using a commercial electrode.

Strains capable of producing N_2_O were also incubated as described above, but with NO_3_^−^ as electron acceptor (NaNO_3_ at a final concentration of 5 mM), and biomass and pH determination were performed as described above. Gas measurements were done with and without 10% C_2_H_2_ gas in the headspace^55^ to verify whether or not they were capable of reducing N_2_O into N_2_.

### Fungal N_2_O production in sterile soil

The physical and chemical parameters of the three soils used in this study are described in Table 1. Triplicate samples were collected from the top 10 cm of three different soils, sieved to <2 mm and sterilized by γ -radiation (35 kGy; Conservatome, Dagneux, France).

Denitrifying strains were incubated with liquid malt medium (400 mL) in 1 L flasks at 25°C, 120 rpm for 7 to 14 days so that enough biomass was obtained. Liquid cultures were centrifuged (12,000 rpm for 10 min) in 50 mL tubes and washed twice with 30 mL of sterile physiological water (0.9% NaCl). For each soil, fungal pellets were resuspended with sterile water and inoculated into two series of triplicate microcosms containing the γ-ray sterilized soil (5 g in 147 mL sterile bottles). Soil moisture was adjusted in order to obtain a water holding capacity (WHC) of 60% after the inoculation, and all the inoculated soil microcosms were incubated for 3–4 weeks at room temperature to allow soil colonization by the fungi. After this pre-incubation, 2 mL of NaNO_2_ solution (10 mM) was added and the soil moisture was adjusted at a WHC of 90%. In half of the replicated microcosms (three for each soil), 10 mL of the ambient air in the headspace was replaced with 10 mL C_2_H_2_, and all bottles were incubated at 25°C for one additional week. Production of N_2_O was also monitored in all three sterile soils without inoculation of fungi (negative controls).

### Nitrous oxide emission measurements

Nitrous oxide production by fungal pure culture was determined by analysing the gas samples collected in the headspace gas using a gas chromatograph (TRACE GC Ultra; Thermo) equipped with an electron capture detector (GC-ECD).

In the inoculated soil microcosms, gas samples were collected 3 times (2, 4, and 7 days after NO_2_ addition) from the headspace and were analysed with GC-ECD to determine the N_2_O concentrations. The N_2_O concentrations were analysed by ANOVA using the GLM procedure in SAS^56^. For the liquid medium culture experiment, strain and substrate (NO_2_ or NO_3_) were used for the fixed effect. For the sterilized soil incubation experiment, strain and soil were used for the fixed effect and strain*soil was included in the model. Tukey's multiple comparisons test was used to separate the means. The significance level was 0.05.

### Nitrous oxide isotopic signature measurements

Ten ml of each headspace gas sample was taken at the end of the incubation experiments and stored in a pre-evacuated vial. The samples were then introduced into a gas chromatograph-isotope ratio mass spectrometer (GC-IRMS) (MAT 252; Thermo Fisher Scientific K.K., Yokohama, Japan) system as described elsewhere to measure the N_2_O isotopomer ratios^57^. Site-specific N isotope analysis in N_2_O was conducted using ion detectors that had been modified for the mass analysis of fragment ions of N_2_O (NO^+^) containing N atoms in the centre positions of N_2_O molecules, whereas the bulk (average) N and oxygen isotope ratios were determined from molecular ions^58^. Pure N_2_O (purity > 99.999%; Showa Denko K.K., Japan) was calibrated with international standards and used as a working standard for the isotopomer ratios. The notation of the isotopomer ratios is shown below. The measurement precision was typically better than 0.1‰ for *δ*^15^N^bulk^ and *δ*^18^O, and better than 0.5‰ for *δ*^15^N^α^ and *δ*^15^N^β^.
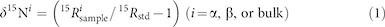

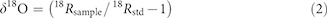


Here, ^15^*R*^α^ and ^15^*R*^β^ respectively represent the ^15^N/^14^N ratios at the centre and end sites of the nitrogen atoms; ^15^*R*^bulk^ and ^18^*R* respectively indicate the average isotope ratios for ^15^N/^14^N and ^18^O/^16^O. The subscripts “sample” and “std” respectively indicate the isotope ratios for the sample and the standard, atmospheric N_2_ for N and Vienna Standard Mean Ocean Water (V-SMOW) for O. We also define the ^15^N site preference (hereinafter SP) as an illustrative parameter of the intramolecular distribution of ^15^N:
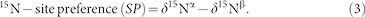
The N_2_O concentration was measured simultaneously with the isotopomer ratios by comparing the peak area of the major ion (mass 44 and 30 in molecular ion analysis and fragment ion analysis, respectively) obtained with the sample gas and with a reference gas (349 nL/L N_2_O in Air; Japan Fine Products Co., Ltd.)^57^.

### Primer design

Full-length *nirK* nucleotide sequences of fungal genomes were obtained from the Functional Gene Pipeline public database (http://fungene.cme.msu.edu//index.spr). These sequences were aligned using MEGA in order to design fungal specific −*nirK* primers. The primer sequences were as follows: EunirK-F1 (5′-GGB AAY CCI CAY AAY ATC GA-3′) and EunirK-R2 (5′-GGI CCI GCR TTS CCR AAG AA-3′).

### DNA extraction, and PCR amplification of the nirK gene and ITS from positive strains

DNA extraction from the denitrifying fungal cultures was performed using a commercially available DNA extraction kit, DNeasy® Plant Maxi (QIAGEN). The extraction was performed according to the manufacturer's instructions. The purified DNA samples were stored at −20°C until further analysis.

The PCR protocol for the *nirK* gene was as follows: 10 min at 94°C and 40 cycles consisting of 1 min at 94°C, 30 s at 53°C, and 1 m at 72°C. For amplification of the fungi Internal Transcribed Spacer (ITS) region, the primer sets ITS5 (5′-TCC TCC GCT TAT TGA TAT GC-3′) and ITS4 (5′-GGA AGT AAA AGT CGT AAC AAG G-3′) were used^59^. The PCR protocol was as follows: 10 min at 95°C and 35 cycles consisting of 95°C for 15 s, 30 s at 52°C, and 72°C for 1.5 s. These amplicons were purified and sequenced with the BigDye Terminator version 3.1 cycle sequencing kit (Applied Biosystems) and the ABI Prism 3100 genetic analyser. The nucleotide sequence of the ITS regions of other fungi and bacterial *nirK* (both class 1 and 2) amino acid sequences were also obtained from the database described above, and the phylogenetic tree was constructed based on the maximum likelihood method using CLUSTALW. Congruence between the *ITS* and *nirK* phylogenies was graphically illustrated using the *cophyloplot* function from the “ape” R package^60^.

## Author Contributions

L.P. and C.S. designed the experiments. K.M., A.S. and L.P. wrote the paper. K.M., C.H., M.C.B. and F.B. screened the fungal isolates. V.E.H. verified the fungal isolates. K.M., S.T. and N.Y. worked on the isotopic experiments.

## Additional Information

Accession codes: All sequence data were submitted to DDBJ under accession numbers AB904794-AB904830.

## Supplementary Material

Supplementary Information Supplementary Information

## Figures and Tables

**Table 1:** Soil characteristics and properties across a range of ecosystem types

Country	Description	Clay	Loam	Sand	Water content	WHC		Total-C	Total-N		OM
		%	%	%	g g^−1^ soil DW	g g^−1^ soil DW	pH	g kg^−1^ soil DW	g kg^−1^ soil DW	C:N ratio	g kg^−1^ soil DW
France	Non-irrigated arable land	18.4	66.4	15.1	0.11	0.50	6.6	10.2	0.97	10.6	17.7
The Netherlands	Permanent Grassland	3.4	2.7	93.8	0.17	0.44	5.1	20.3	1.25	16.3	35.1
Sweden	Coniferous forest	21.4	15.9	62.7	1.51	2.54	4.3	253.0	14.2	17.8	438.0

WHC: water holding capacity; OM: organic matter; DW: dry weight.

## References

[b1] Davidson E. A., Rogers J. & Whitman W. Fluxes of nitrous oxide and nitric oxide from terrestrial ecosystems. In: Microbial production and consumption of greenhouse gases: methane, nitrogen oxides, and halomethanes. (eds. Rogers J. E., , Whitman W. B., eds. ) 219–235. (American Society for Microbiology, 1991).

[b2] Davidson E. The contribution of manure and fertilizer nitrogen to atmospheric nitrous oxide since 1860. Nat. Geosci. 2, 659–662 (2009).

[b3] Forster P. *et al.* Changes in Atmospheric Constituents and in Radiative Forcing. In: Climate Change 2007: The Physical Science Basis. (eds. Solomon S., *et al.*) Ch.2, 130–234. (Cambridge University Press, 2007).

[b4] Ravishankara A., Daniel J. & Portmann R. Nitrous oxide (N_2_O): the dominant ozone-depleting substance emitted in the 21st century. Science 326, 123–125 (2009). 19713491 10.1126/science.1176985

[b5] Hooper A. B. & Terry K. Hydroxylamine oxidoreductase of *Nitrosomonas*: Production of nitric oxide from hydroxylamine. BBA-Enzymol. 571, 12–20 (1979). 10.1016/0005-2744(79)90220-1 497235

[b6] Zumft W. Cell biology and molecular basis of denitrification. Microbiol. Mol. Biol. Rev. 61, 533–616 (1997). 9409151 10.1128/mmbr.61.4.533-616.1997 PMC232623

[b7] Thomson A. J., Giannopoulos G., Pretty J., Baggs E. M. & Richardson D. J. Biological sources and sinks of nitrous oxide and strategies to mitigate emissions. Phil. Trans. R. Soc. B: Biol. Sci. 367, 1157–1168 (2012). 10.1098/rstb.2011.0415 PMC3306631 22451101

[b8] Ostrom N. *et al.* Isotopologue data reveal bacterial denitrification as the primary source of N_2_O during a high flux event following cultivation of a native temperate grassland. Soil Biol. Biochem. 42, 499–506 (2010).

[b9] Philippot L., Hallin S. & Schloter M. Ecology of denitrifying prokaryotes in agricultural soil. Adv. Agron. 96, 249–305 (2007).

[b10] Shoun H., Fushinobu S., Jiang L., Kim S. W. & Wakagi T. Fungal denitrification and nitric oxide reductase cytochrome P450nor. Phil. Trans. R. Soc. B: Biol. Sci. 367, 1186–1194 (2012). 10.1098/rstb.2011.0335 PMC3306627 22451104

[b11] Risgaard-Petersen N. *et al.* Evidence for complete denitrification in a benthic foraminifer. Nature 443, 93–96 (2006). 16957731 10.1038/nature05070

[b12] Piña-Ochoa E. *et al.* Widespread occurrence of nitrate storage and denitrification among *Foraminifera* and *Gromiida*. Proc. Natl. Acad. Sci. 107, 1148–1153 (2010). 20080540 10.1073/pnas.0908440107 PMC2824274

[b13] Hayatsu M., Tago K. & Saito M. Various players in the nitrogen cycle: diversity and functions of the microorganisms involved in nitrification and denitrification. Soil Sci. Plant Nutr. 54, 33–45 (2008).

[b14] Shoun H., Kim D. H., Uchiyama H. & Sugiyama J. Denitrification by fungi. FEMS Microbiol. Lett. 94, 277–281 (1992). 1426992 10.1016/0378-1097(92)90643-3

[b15] Herold M. B., Baggs E. M. & Daniell T. J. Fungal and bacterial denitrification are differently affected by long-term pH amendment and cultivation of arable soil. Soil Biol. Biochem. 54, 25–35 (2012).

[b16] Laughlin R. J., Rütting T., Müller C., Watson C. J. & Stevens R. J. Effect of acetate on soil respiration, N_2_O emissions and gross N transformations related to fungi and bacteria in a grassland soil. Appl. Soil Ecol. 42, 25–30 (2009).

[b17] Marusenko Y., Huber D. P. & Hall S. J. Fungi mediate nitrous oxide production but not ammonia oxidation in aridland soils of the southwestern US. Soil Biol. Biochem. 63, 24–36 (2013).

[b18] Lundell T. K., Mäkelä M. R. & Hildén K. Lignin‐modifying enzymes in filamentous basidiomycetes–ecological, functional and phylogenetic review. J. Bas. Microbiol. 50, 5–20 (2010). 10.1002/jobm.200900338 20175122

[b19] Alabouvette C., Olivain C., Migheli Q. & Steinberg C. Microbiological control of soil‐borne phytopathogenic fungi with special emphasis on wilt‐inducing *Fusarium oxysporum*. New Phytol. 184, 529–544 (2009). 19761494 10.1111/j.1469-8137.2009.03014.x

[b20] Tsuruta S. *et al.* Denitrification by yeasts and occurrence of cytochrome P450nor in *Trichosporon cutaneum*. FEMS Microbiol. Lett. 168, 105–110 (1998). 9812370 10.1111/j.1574-6968.1998.tb13262.x

[b21] Zhou Z., Takaya N., Sakairi M. A. C. & Shoun H. Oxygen requirement for denitrification by the fungus *Fusarium oxysporum*. Arch. Microbiol. 175, 19–25 (2001). 11271416 10.1007/s002030000231

[b22] Daum D. & Schenk M. K. Influence of nutrient solution pH on N_2_O and N_2_ emissions from a soilless culture system. Plant Soil 203, 279–288 (1998).

[b23] Takaya N. Dissimilatory nitrate reduction metabolisms and their control in fungi. J. Biosci. Bioeng. 94, 506–510 (2002). 16233342 10.1016/s1389-1723(02)80187-6

[b24] Shoun H. & Tanimoto T. Denitrification by the fungus *Fusarium oxysporum* and involvement of cytochrome P-450 in the respiratory nitrite reduction. J. Biol. Chem. 266, 11078–11082 (1991). 2040619

[b25] Throback I. N., Enwall K., Jarvis A. & Hallin S. Reassessing PCR primers targeting *nirS*, *nirK* and *nosZ* genes for community surveys of denitrifying bacteria with DGGE. FEMS Microbiol. Ecol. 49, 401–417 (2004). 19712290 10.1016/j.femsec.2004.04.011

[b26] Jones C. M., Graf D. R. H., Bru D., Philippot L. & Hallin S. The unaccounted yet abundant nitrous oxide-reducing microbial community: a potential nitrous oxide sink. ISME J. 7, 417–426 (2013). 23151640 10.1038/ismej.2012.125 PMC3554408

[b27] Cheneby D. *et al.* Denitrifying bacteria in bulk and maize-rhizospheric soil: diversity and N_2_O-reducing abilities. Canad. J. Microbiol. 50, 469–474 (2004). 15381970 10.1139/w04-037

[b28] Bru D. *et al.* Determinants of the distribution of nitrogen-cycling microbial communities at the landscape scale. ISME J. 5, 532–542 (2010). 20703315 10.1038/ismej.2010.130 PMC3105713

[b29] Jones C. M., Stres B., Rosenquist M. & Hallin S. Phylogenetic analysis of nitrite, nitric oxide, and nitrous oxide respiratory enzymes reveal a complex evolutionary history for denitrification. Mol. Biol. Evol. 25, 1955–1966 (2008). 18614527 10.1093/molbev/msn146

[b30] Mahne I. & Tiedje J. M. Criteria and methodology for identifying respiratory denitrifiers. Appl. Environ. Microbiol. 61, 1110–1115 (1995). 16534960 10.1128/aem.61.3.1110-1115.1995 PMC1388392

[b31] Jones C. M. *et al.* Phenotypic and genotypic heterogeneity among closely related soil‐borne N_2_^-^ and N_2_O^-^ producing *Bacillus* isolates harboring the *nosZ* gene. FEMS Microbiol. Ecol. 76, 541–552 (2011). 21348884 10.1111/j.1574-6941.2011.01071.x

[b32] Klaubauf S. *et al.* Molecular diversity of fungal communities in agricultural soils from Lower Austria. Fung. Div. 44, 65–75 (2010). 10.1007/s13225-010-0053-1 PMC3688302 23794962

[b33] Orgiazzi A. *et al.* Unravelling soil fungal communities from different Mediterranean land-use backgrounds. PLoS One 7, e34847 (2012). 22536336 10.1371/journal.pone.0034847 PMC3335027

[b34] Rudolph N. *et al.* Dynamic oxygen mapping in the root zone by fluorescence dye imaging combined with neutron radiography. J. Soils *Sed.*12, 63–74 (2012).

[b35] Corrales Escobosa A. R. *et al.* Fusarium oxysporum Adh1 has dual fermentative and oxidative functions and is involved in fungal virulence in tomato plants. Fung. Genet. Biol. 48, 886–895 (2011). 10.1016/j.fgb.2011.06.004 21704720

[b36] Yoshida N. & Toyoda S. Constraining the atmospheric N_2_O budget from intramolecular site preference in N_2_O isotopomers. Nature 405, 330–334 (2000). 10830958 10.1038/35012558

[b37] Sutka R., Adams G., Ostrom N. & Ostrom P. Isotopologue fractionation during N_2_O production by fungal denitrification. Rapid Commun. Mass Spectrom. 22, 3989–3996 (2008). 19016253 10.1002/rcm.3820

[b38] Samarkin V. *et al.* Abiotic nitrous oxide emission from the hypersaline Don Juan Pond in Antarctica. Nat. Geosci. 3, 341–344 (2010).

[b39] Sutka R. L. *et al.* Distinguishing nitrous oxide production from nitrification and denitrification on the basis of isotopomer abundances. Appl. Environ. Microbiol. 72, 638–644 (2006). 16391101 10.1128/AEM.72.1.638-644.2006 PMC1352222

[b40] Jung M.-Y. *et al.* Isotopic signatures of N_2_O produced by ammonia-oxidizing archaea from soils. ISME J. 8, 1115–1125 (2013). 24225887 10.1038/ismej.2013.205 PMC3996685

[b41] Santoro A. E., Buchwald C., McIlvin M. R. & Casciotti K. L. Isotopic signature of N_2_O produced by marine ammonia-oxidizing archaea. Science 333, 1282–1285 (2011). 21798895 10.1126/science.1208239

[b42] Wei W. *et al.* N_2_O emission from cropland field soil through fungal denitrification after surface applications of organic fertilizer. Soil Biol. Biochem. 69, 157–167 (2014).

[b43] Enwall K., Philippot L. & Hallin S. Activity and composition of the denitrifying bacterial community respond differently to long-term fertilization. Appl. Environ. Microbiol. 71, 8335–8343 (2005). 16332820 10.1128/AEM.71.12.8335-8343.2005 PMC1317341

[b44] Braker G., Schwarz J. & Conrad R. Influence of temperature on the composition and activity of denitrifying soil communities. FEMS Microbiol. Ecol. 73, 134–148 (2010). 20455938 10.1111/j.1574-6941.2010.00884.x

[b45] Orlando J., Carú M., Pommerenke B. & Braker G. Diversity and activity of denitrifiers of Chilean arid soil ecosystems. Front. Microbiol. 3, 101 (2012). 22493591 10.3389/fmicb.2012.00101 PMC3319911

[b46] Lauber C. L., Strickland M. S., Bradford M. A. & Fierer N. The influence of soil properties on the structure of bacterial and fungal communities across land-use types. Soil Biol. Biochem. 40, 2407–2415 (2008).

[b47] Wubet T. *et al.* Differences in soil fungal communities between European beech (*Fagus sylvatica L*.) dominated forests are related to soil and understory vegetation. PloS One 7, e47500 (2012). 23094057 10.1371/journal.pone.0047500 PMC3475711

[b48] Joergensen R. G. & Wichern F. Quantitative assessment of the fungal contribution to microbial tissue in soil. Soil Biol. Biochem. 40, 2977–2991 (2008).

[b49] Braker G., Zhou J., Wu L., Devol A. H. & Tiedje J. M. Nitrite reductase genes (*nirK* and *nirS*) as functional markers to investigate diversity of denitrifying bacteria in pacific northwest marine sediment communities. Appl. Environ. Microbiol. 66, 2096–2104 (2000). 10788387 10.1128/aem.66.5.2096-2104.2000 PMC101460

[b50] Kim S.-W., Fushinobu S., Zhou S., Wakagi T. & Shoun H. Eukaryotic *nirK* genes encoding copper-containing nitrite reductase: originating from the protomitochondrion? Appl. Environ. Microbiol. 75, 2652–2658 (2009). 19270125 10.1128/AEM.02536-08 PMC2681669

[b51] Liu B., Mao Y., Bergaust L., Bakken L. R. & Frostegård Å. Strains in the genus *Thauera* exhibit remarkably different denitrification regulatory phenotypes. Environ. Microbiol. 15, 2816–2828 (2013). 23663391 10.1111/1462-2920.12142

[b52] Edel V., Steinberg C., Gautheron N., Recorbet G. & Alabouvette C. Genetic diversity of *Fusarium oxysporum* populations isolated from different soils in France. FEMS Microbiol. Ecol. 36, 61–71 (2001). 11377774 10.1111/j.1574-6941.2001.tb00826.x

[b53] Nakanishi Y. *et al.* A eukaryotic copper-containing nitrite reductase derived from a NirK homolog gene of *Aspergillus oryzae*. Biosci. Biotechnol. Biochem. 74, 984–991 (2010). 20460712 10.1271/bbb.90844

[b54] Usuda K., Toritsuka N., Matsuo Y., Kim D. H. & Shoun H. Denitrification by the fungus *Cylindrocarpon tonkinense*: anaerobic cell growth and two isozyme forms of cytochrome P-450nor. Appl. Environ. Microbiol. 61, 883–889 (1995). 7793922 10.1128/aem.61.3.883-889.1995 PMC167353

[b55] Yoshinari T. & Knowles R. Acetylene inhibition of nitrous oxide reduction by denitrifying bacteria. Biochem. Biophys. Res. Commun. 69, 705–710 (1976). 817722 10.1016/0006-291x(76)90932-3

[b56] SAS Institute Inc. . SAS/STAT 9.1 user's guide. (SAS Institute Inc., 2004).

[b57] Toyoda S., Mutobe H., Yamagishi H., Yoshida N. & Tanji Y. Fractionation of N_2_O isotopomers during production by denitrifier. Soil Biol. Biochem. 37, 1535–1545 (2005).

[b58] Toyoda S. & Yoshida N. Determination of nitrogen isotopomers of nitrous oxide on a modified isotope ratio mass spectrometer. Anal. Chem. 71, 4711–4718 (1999).

[b59] Schoch C. L. *et al.* Nuclear ribosomal internal transcribed spacer (ITS) region as a universal DNA barcode marker for Fungi. Proc. Natl. Acad. Sci. 109, 6241–6246 (2012). 22454494 10.1073/pnas.1117018109 PMC3341068

[b60] Paradis E., Claude J. & Strimmer K. APE: analyses of phylogenetics and evolution in R language. Bioinformatics 20, 289–290 (2004). 14734327 10.1093/bioinformatics/btg412

