# Supplementary material for: N2O production, a widespread trait in fungi
Source: Sci Rep. 2015 Apr 20;5:9697. doi: 10.1038/srep09697 (PMC4403702; doi:10.1038/srep09697)
Supplement: Supplementary Information [file srep09697-s1.pdf]

Supplemental information

Manuscript title: N_2O production, a widespread trait in fungi

Author list: Koki Maeda[1, 2*], Aymé Spor[2], Véronique Edel-Hermann[2], Cécile Heraud[2], Marie-Christine Breuil[2], Florian Bizouard[2], Sakae Toyoda[3], Naohiro Yoshida[3,4,5], Christian Steinberg[2] and Laurent Philippot

Maeda et al., Fig. S1

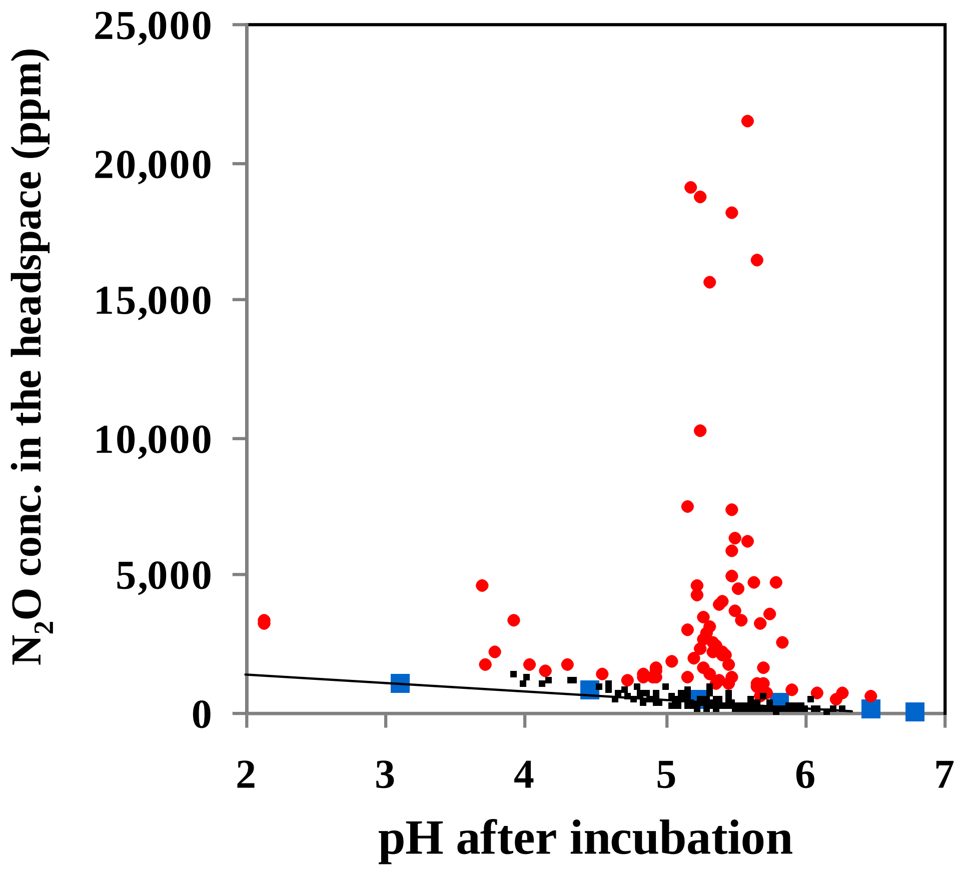

Figure S1: Relationship between N_2O concentrations and pH after incubation. Blue rectangles indicate the negative controls without fungi cultures. Red circles indicate the positive strains and black dots indicate the negative strains.

Maeda et al., Fig. S2

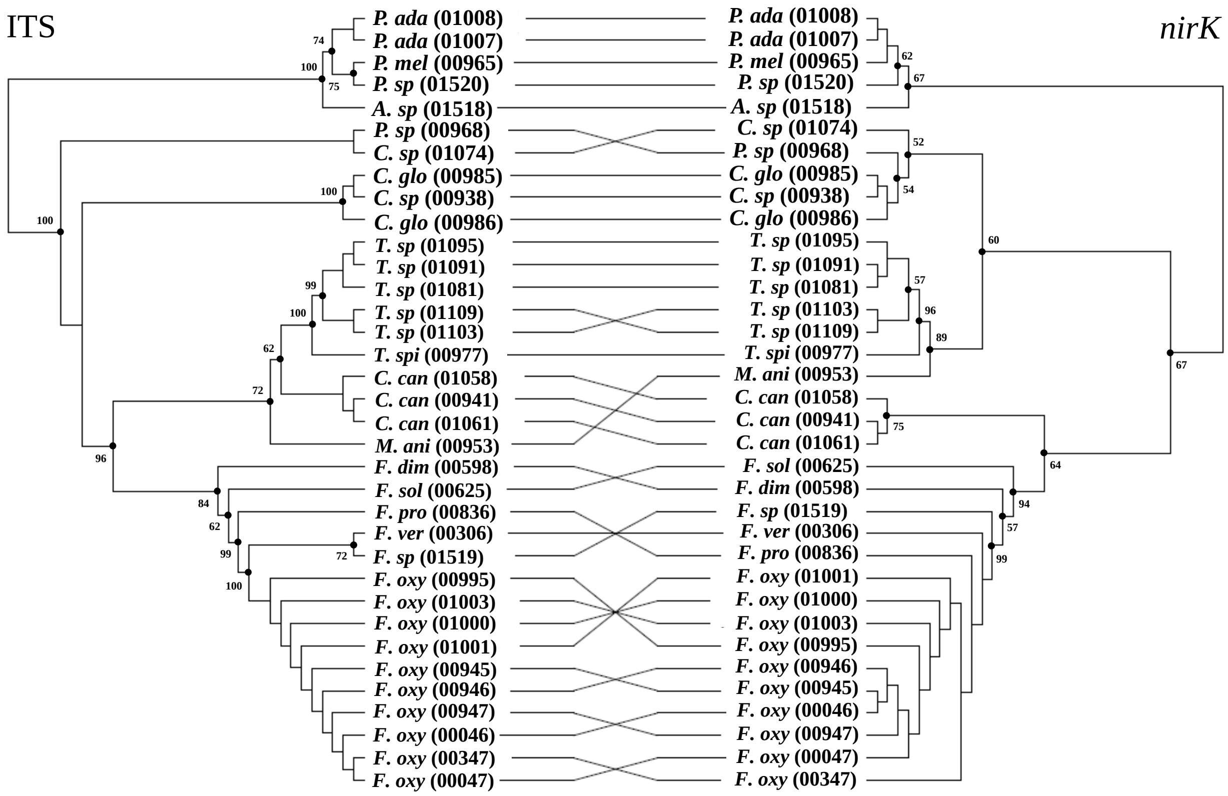

Figure S2: Congruence between the ITS (left) and nirK (right) phylogenies. This plot was generated using the cophyloplot function from the "ape" R package. Nodes with >50% (●) bootstrap support are indicated. The numbers in parentheses are MIAE numbers. Abbreviated strain names are used. The full names of the strains are given in Table S1.

Maeda et al., Table S1

Table S1 $\delta^{15}N_{bulk}$, $\delta^{18}O$ and site preference (SP) values of produced N_2O

Strain Name	MIAE No.	SP (‰)		$\delta^{15}N_{bulk}$ (‰)		$\delta^{18}O$ (‰)	
		average	sd	average	sd	average	sd
Absidia glauca	01056	**23.9**	3.0	**1.6**	0.1	**31.4**	0.3
Aspergillus sp.	01518	**24.7**	5.6	**-16.2**	1.6	**29.6**	2.4
Chaetomium sp.	00938	**35.7**	1.6	**-0.7**	1.9	**42.5**	0.1
Chaetomium sp.	00985	**31.8**	0.3	**-1.6**	2.6	**43.6**	2.4
Chaetomium sp.	00986	**33.4**	2.2	**2.4**	1.5	**45.0**	1.2
Chaetomiaceae	00990	**28.0**	2.8	**-4.1**	1.7	**32.9**	1.8
Chloridium sp.	01074	**30.3**	0.6	**-8.0**	0.2	**33.6**	2.1
Chloridium virescens	00939	**34.0**	7.0	**-8.1**	0.3	**35.6**	1.1
C. virescens	01069	**32.3**	2.3	**-8.7**	0.1	**33.9**	0.1
Clonostachys candelabrum	00941	**30.7**	3.7	**-19.2**	1.8	**41.8**	2.4
C. candelabrum	01058	**34.6**	3.9	**-6.6**	0.5	**41.4**	0.8
C. candelabrum	01061	**34.5**	1.9	**-7.8**	0.3	**38.9**	0.0
Colletotrichum coccodes	01515	**31.2**	2.3	**-21.9**	0.3	**36.7**	0.2
Fusarium acuminatum	00244	**33.2**	1.2	**-8.6**	0.6	**38.4**	0.7
F. ticinctum species complex	00285	**30.7**	0.9	**-6.5**	0.7	**38.2**	0.6
F. avenaceum	00286	**36.3**	1.8	**-6.7**	0.5	**38.6**	0.6
F. dimerum	00598	**27.9**	1.9	**-5.4**	0.4	**36.5**	0.1
F. proliferatum	00836	**30.0**	1.1	**-6.7**	0.2	**30.4**	2.3
F. sambucinum	00301	**36.7**	2.2	**-9.4**	0.2	**35.8**	0.1
F. solani	00625	**30.0**	1.6	**-4.0**	0.0	**33.2**	0.0
F. oxysporum f. sp. Lini	00347	**30.3**	2.0	**-1.8**	1.4	**37.5**	0.4
F. oxysporum	00046	**35.7**	2.7	**0.9**	3.6	**36.6**	1.9
F. oxysporum	00047	**34.1**	0.7	**5.9**	2.5	**35.9**	0.4
F. oxysporum	00945	**31.4**	5.0	**-3.2**	1.0	**34.3**	2.9
F. oxysporum	00946	**32.7**	0.6	**-5.5**	0.6	**34.8**	0.5
F. oxysporum	00947	**23.0**	2.8	**-3.5**	0.2	**29.6**	0.2
F. oxysporum	00948	**34.4**	3.1	**-1.7**	2.3	**37.3**	1.6
F. oxysporum	00996	**33.4**	5.0	**0.8**	2.5	**37.6**	1.5
F. oxysporum	00991	**32.2**	2.0	**-5.4**	0.8	**35.5**	0.2
F. oxysporum	00994	**26.2**	7.1	**-2.9**	1.5	**34.1**	2.5
F. oxysporum	00998	**30.5**	1.8	**-4.0**	1.0	**32.0**	0.6
F. oxysporum	01000	**28.7**	1.3	**-2.2**	0.1	**33.0**	0.5
F. oxysporum	01001	**32.7**	0.8	**-5.9**	1.4	**36.2**	1.9
F. oxysporum	01003	**35.1**	0.0	**0.8**	0.7	**39.0**	0.8
Fusarium sp.	01519	**30.4**	5.1	**-3.1**	11.5	**34.2**	4.0
F. verticillioides	00306	**27.2**	4.8	**-4.7**	0.8	**32.5**	3.9
Leptosphaeria sp.	01060	**29.0**	7.2	**-11.9**	0.1	**37.5**	0.1
Metarhizium anisopliae	00953	**30.5**	0.3	**-8.8**	0.3	**36.1**	0.0
Penicillium adametzii	01007	**20.6**	2.4	**1.7**	1.2	**30.6**	0.8
P. adametzii	01008	**21.2**	0.8	**-0.4**	1.8	**32.2**	1.2
P. melinii	01009	**19.2**	1.7	**3.3**	0.3	**31.5**	0.4
P. melinii	00965	**15.8**	2.6	**2.5**	0.8	**29.4**	0.9
Penicillium sp.	01010	**22.7**	3.4	**1.3**	0.9	**31.7**	0.6
Penicillium sp.	01520	**19.0**	2.2	**1.1**	0.6	**31.9**	1.1
Phialocephala sp.	00968	**34.0**	1.7	**-21.8**	1.9	**36.4**	3.8
Trichoderma harzianum	00042	**30.0**	4.0	**-20.2**	0.6	**40.1**	2.1
T. harzianum (anamorph)	01011	**31.7**	1.5	**-8.3**	1.5	**38.5**	1.5
T. harzianum (anamorph)	01087	**33.4**	0.6	**-8.1**	0.4	**36.8**	0.3
T. harzianum (anamorph)	01113	**32.7**	1.0	**-9.0**	0.2	**36.4**	0.0
T. spirale	00977	**31.5**	0.2	**-8.9**	0.1	**36.3**	0.6
T. tomentosum	00031	**32.7**	4.7	**-9.5**	0.1	**37.1**	0.0
Trichoderma sp.	01015	**16.6**	1.4	**1.3**	0.3	**26.3**	0.9
Trichoderma sp.	01077	**31.6**	0.6	**-8.0**	0.0	**36.3**	0.2
Trichoderma sp.	01080	**32.0**	0.1	**-8.5**	0.3	**36.7**	0.1
Trichoderma sp.	01081	**32.3**	1.5	**-8.7**	0.3	**36.1**	0.1
Trichoderma sp.	01091	**31.5**	1.6	**-8.1**	0.4	**34.3**	0.5
Trichoderma sp.	01092	**31.2**	1.4	**-8.5**	0.1	**36.9**	0.1
Trichoderma sp.	01093	**35.1**	2.4	**-8.8**	0.3	**35.7**	0.0
Trichoderma sp.	01094	**27.1**	2.1	**-3.5**	1.4	**32.3**	0.8
Trichoderma sp.	01095	**35.1**	1.6	**-8.7**	0.2	**35.6**	0.7
Trichoderma sp.	01102	**24.6**	0.4	**-3.9**	4.8	**31.4**	1.9
Trichoderma sp.	01103	**34.6**	3.7	**-9.9**	0.1	**35.8**	0.3
Trichoderma sp.	01106	**29.2**	0.4	**-5.8**	0.4	**33.8**	0.8
Trichoderma sp.	01107	**31.7**	0.9	**-7.6**	0.9	**36.8**	1.0
Trichoderma sp.	01109	**32.1**	0.7	**-7.7**	0.3	**33.5**	0.2
Trichoderma sp.	01115	**31.9**	0.7	**-7.8**	0.2	**34.2**	0.2
T. virens	00952	**23.9**	3.8	**0.4**	1.9	**31.2**	0.4

MIAE: Microorganismes d'Intérêt Agro-Environnemental, UMR Agroécologie, INRA, Dijon, Franc

Table S2	List of the strains tested				
MIAE	Other code	Identification	Phylum	Class	Order
00031	T32	*Trichoderma tomentosum*	Ascomycota	Sordariomycetes	Hypocreales
00042	T45	*Trichoderma harzianum*	Ascomycota	Sordariomycetes	Hypocreales
00046	Fol8	*Fusarium oxysporum f.* sp. *lycopersici*	Ascomycota	Sordariomycetes	Hypocreales
00047	Fo47	*Fusarium oxysporum*	Ascomycota	Sordariomycetes	Hypocreales
00050	FEQ1	*Fusarium equiseti*	Ascomycota	Sordariomycetes	Hypocreales
00068	FSUB02	*Fusarium subglutinans*	Ascomycota	Sordariomycetes	Hypocreales
00149	0628-001 1 B PDA	*Penicillium polonicum*	Ascomycota	Eurotiomycetes	Eurotiales
00174	0629-024 2 WA	*Microdochium bolleyi*	Ascomycota	Sordariomycetes	Xylariales
00244	FAC01	*Fusarium acuminatum*	Ascomycota	Sordariomycetes	Hypocreales
00285	FAR01	*Fusarium ticinctum* species complex	Ascomycota	Sordariomycetes	Hypocreales
00286	FAV05	*Fusarium avenaceum*	Ascomycota	Sordariomycetes	Hypocreales
00287	FCHL02	*Fusarium chlamydosporum*	Ascomycota	Sordariomycetes	Hypocreales
00299	FPO01	*Fusarium poae*	Ascomycota	Sordariomycetes	Hypocreales
00301	FSA02	*Fusarium sambucinum*	Ascomycota	Sordariomycetes	Hypocreales
00304	FSPO01	*Fusarium sporotrichioides*	Ascomycota	Sordariomycetes	Hypocreales
00306	FVER03	*Fusarium verticillioides*	Ascomycota	Sordariomycetes	Hypocreales
00312	FLANG03	*Fusarium langsethiae*	Ascomycota	Sordariomycetes	Hypocreales
00315	GGT	*Gaeumannomyces graminis var. tritici*	Ascomycota	Sordariomycetes	Magnaporthales
00316	MNIV01	*Microdochium nivale*	Ascomycota	Sordariomycetes	Xylariales
00322	T4	*Botrytis cinerea*	Ascomycota	Leotiomycetes	Helotiales
00329	Bd90	*Botrytis cinerea*	Ascomycota	Leotiomycetes	Helotiales
00344	VERT03	*Verticillium chlamydosporum*	Ascomycota	Sordariomycetes	Hypocreales
00345	VERT02	*Verticillium lecanii*	Ascomycota	Sordariomycetes	Hypocreales
00346	VERT04	*Verticillium nigricans*	Ascomycota	Sordariomycetes	Hypocreales
00347	Foln3	*Fusarium oxysporum f.* sp. *lini*	Ascomycota	Sordariomycetes	Hypocreales
00376	BASF270	*Fusarium graminearum*	Ascomycota	Sordariomycetes	Hypocreales
00382	0610-001 1 C β PDA	*Penicillium freii*	Ascomycota	Eurotiomycetes	Eurotiales
00456		*Fusarium pseudograminearum*	Ascomycota	Sordariomycetes	Hypocreales
00598	IHEM10066	*Fusarium dimerum*	Ascomycota	Sordariomycetes	Hypocreales
00625	FS59	*Fusarium solani*	Ascomycota	Sordariomycetes	Hypocreales
00690	FRED1	*Fusarium redolens*	Ascomycota	Sordariomycetes	Hypocreales
00836	FPRO3	*Fusarium proliferatum*	Ascomycota	Sordariomycetes	Hypocreales
	0629-002 J 4 A β PDA	*Epicoccum nigrum*	Ascomycota	Dothideomycetes	mitosporic Dothideomycetes
	0629-002 J 4 C PDA	*Alternaria citri*	Ascomycota	Dothideomycetes	Pleosporales
	0722-001 1 D PDA	*Penicillium brasilianum*	Ascomycota	Eurotiomycetes	Eurotiales
	0628-019 2 WA	*Cephalosporium* sp.	Ascomycota	Sordariomycetes	Hypocreales
	0628-019 3 β WA	*Clonostachys rosea*	Ascomycota	Sordariomycetes	Hypocreales
	0629-033 2 A β WA	*Gliocladium* sp.	Ascomycota	Sordariomycetes	Hypocreales
00864	JCM11502	*Fusarium oxysporum*	Ascomycota	Sordariomycetes	Hypocreales
00871	NBRC30561	*Cylindrocarpon lichenicola*	Ascomycota	Sordariomycetes	Hypocreales
00872	NBRC100959	*Aspergillus oryzae*	Ascomycota	Eurotiomycetes	Eurotiales
00938	SYS609	*Chaetomium* sp.	Ascomycota	Sordariomycetes	Sordariales
00939	SYS473	*Chloridium virescens*	Ascomycota	Sordariomycetes	Chaetosphaeriales
00940	SYS068	*Cladosporium cladosporioides*	Ascomycota	Dothideomycetes	Capnodiales
00941	SYS300	*Clonostachys candelabrum*	Ascomycota	Sordariomycetes	Hypocreales
00943	SYS681	*Davidiella tassiana*	Ascomycota	Dothideomycetes	Capnodiales
00945	SYS026	*Fusarium oxysporum*	Ascomycota	Sordariomycetes	Hypocreales
00946	SYS158	*Fusarium oxysporum*	Ascomycota	Sordariomycetes	Hypocreales
00947	SYS400	*Fusarium oxysporum*	Ascomycota	Sordariomycetes	Hypocreales
00948	SYS636	*Fusarium oxysporum*	Ascomycota	Sordariomycetes	Hypocreales
00949	SYS350	*Geomyces destructans*	Ascomycota	Leotiomycetes	Leotiomycetes incertae sedis
00950	SYS331	*Hypocrea koningii*	Ascomycota	Sordariomycetes	Hypocreales
00951	SYS129	*Hypocrea lixii*	Ascomycota	Sordariomycetes	Hypocreales
00952	SYS607	*Trichoderma virens*	Ascomycota	Sordariomycetes	Hypocreales
00953	SYS540	*Metarhizium anisopliae*	Ascomycota	Sordariomycetes	Hypocreales
00954	SYS868	*Microsphaeropsis arundinis*	Ascomycota	-	mitosporic Ascomycota
00960	SYS418	*Penicillium adametzii*	Ascomycota	Eurotiomycetes	Eurotiales
00961	SYS570	*Penicillium canescens*	Ascomycota	Eurotiomycetes	Eurotiales
00962	SYS078	*Penicillium citreonigrum*	Ascomycota	Eurotiomycetes	Eurotiales
00963	SYS483	*Penicillium decaturense*	Ascomycota	Eurotiomycetes	Eurotiales
00964	SYS386	*Penicillium fagi*	Ascomycota	Eurotiomycetes	Eurotiales
00965	SYS203	*Penicillium melinii*	Ascomycota	Eurotiomycetes	Eurotiales
00966	SYS846	*Penicillium montanense*	Ascomycota	Eurotiomycetes	Eurotiales
00967	SYS536	*Penicillium pinophilum*	Ascomycota	Eurotiomycetes	Eurotiales
00968	SYS875	*Phialocephala* sp.	Ascomycota	Sordariomycetes	Ophiostomatales
00969	SYS615	*Phoma americana*	Ascomycota	Dothideomycetes	Pleosporales
00970	SYS088	*Phoma pomorum*	Ascomycota	Dothideomycetes	Pleosporales
00971	SYS327	*Pochonia bulbillosa*	Ascomycota	Sordariomycetes	Hypocreales

00973	SYS805	*Trichiderma asperellum*	Ascomycota	Sordariomycetes	Hypocreales
00974	SYS877	*Trichoderma gamsii*	Ascomycota	Sordariomycetes	Hypocreales
00975	SYS029	*Trichoderma hamatum*	Ascomycota	Sordariomycetes	Hypocreales
00976	SYS284	*Trichoderma koningiopsis*	Ascomycota	Sordariomycetes	Hypocreales
00977	SYS624	*Trichoderma spirale*	Ascomycota	Sordariomycetes	Hypocreales
00978	SYS629	*Trichoderma viride*	Ascomycota	Sordariomycetes	Hypocreales
00982	SYS523	*Zalerion varium*	Ascomycota	-	mitosporic Ascomycota
00984	SYS310	*Penicillium griseolum*	Ascomycota	Eurotiomycetes	Eurotiales
00985	SYS610	*Chaetomium* sp.	Ascomycota	Sordariomycetes	Sordariales
00986	SYS538	*Chaetomium* sp.	Ascomycota	Sordariomycetes	Sordariales
00987	SYS051	*Chaetomium piluliferum / Humicola fuscoatra*	Ascomycota	Sordariomycetes	Sordariales
00989	SYS022	*Chaetomium sp / Humicola fuscoatra*	Ascomycota	Sordariomycetes	Sordariales
00990	SYS971	*Chaetomiaceae*	Ascomycota	Sordariomycetes	Sordariales
00991	SYS085	*Fusarium oxysporum*	Ascomycota	Sordariomycetes	Hypocreales
00992	SYS822	*Penicillium citreonigrum*	Ascomycota	Eurotiomycetes	Eurotiales
00993	SYS054	*Fusarium oxysporum*	Ascomycota	Sordariomycetes	Hypocreales
00994	SYS091	*Fusarium oxysporum*	Ascomycota	Sordariomycetes	Hypocreales
00995	SYS125	*Fusarium oxysporum*	Ascomycota	Sordariomycetes	Hypocreales
00996	SYS159	*Fusarium oxysporum*	Ascomycota	Sordariomycetes	Hypocreales
00997	SYS469	*Fusarium oxysporum*	Ascomycota	Sordariomycetes	Hypocreales
00998	SYS500	*Fusarium oxysporum*	Ascomycota	Sordariomycetes	Hypocreales
00999	SYS591	*Fusarium oxysporum*	Ascomycota	Sordariomycetes	Hypocreales
01000	SYS661	*Fusarium oxysporum*	Ascomycota	Sordariomycetes	Hypocreales
01001	SYS795	*Fusarium oxysporum*	Ascomycota	Sordariomycetes	Hypocreales
01002	SYS851	*Fusarium oxysporum*	Ascomycota	Sordariomycetes	Hypocreales
01003	SYS950	*Fusarium oxysporum*	Ascomycota	Sordariomycetes	Hypocreales
01004	SYS417	*Fusarium aethiopicum / Fusarium culmorum / Fusarium vorosii*	Ascomycota	Sordariomycetes	Hypocreales
01005	SYS788	*Chaetomium* sp. / *Humicola* sp.	Ascomycota	Sordariomycetes	Sordariales
01006	SYS146	*Penicillium aculeatum / Penicillium pinophilum*	Ascomycota	Eurotiomycetes	Eurotiales
01007	SYS213	*Penicillium adametzii*	Ascomycota	Eurotiomycetes	Eurotiales
01008	SYS550	*Penicillium adametzii*	Ascomycota	Eurotiomycetes	Eurotiales
01009	SYS577	*Penicillium melinii*	Ascomycota	Eurotiomycetes	Eurotiales
01010	SYS461	*Penicillium* sp.	Ascomycota	Eurotiomycetes	Eurotiales
01011	SYS579	*Trichoderma harzianum* (anamorph)	Ascomycota	Sordariomycetes	Hypocreales
01012	SYS468	*Hypocrea chlorospora*	Ascomycota	Sordariomycetes	Hypocreales
01013	SYS059	*Hypocrea koningii*	Ascomycota	Sordariomycetes	Hypocreales
01014	SYS002	*Hypocrea lixii*	Ascomycota	Sordariomycetes	Hypocreales
01015	SYS239	*Trichoderma koningiopsis / Hypocrea koningii*	Ascomycota	Sordariomycetes	Hypocreales
01047	SYS090	*Phoma americana / Epicoccum americana*	Ascomycota	Dothideomycetes	Pleosporales
01048	SYS204	*Eupenicillium pinetorum / Penicillium montanense*	Ascomycota	Eurotiomycetes	Eurotiales
01050	SYS281	*Leptodontidium* sp.	Ascomycota	mitosporic Ascomycota	mitosporic Ascomycota
01053	SYS368	*Penicillium citreonigrum*	Ascomycota	Eurotiomycetes	Eurotiales
01054	SYS355	*Tolypocladium cylindrosporum*	Ascomycota	Sordariomycetes	Hypocreales
01057	SYS543	*Acremonium strictum*	Ascomycota	Sordariomycetes	Hypocreales
01058	SYS632	*Clonostachys candelabrum*	Ascomycota	Sordariomycetes	Hypocreales
01060	SYS651	*Leptosphaeria* sp.	Ascomycota	Dothideomycetes	Pleosporales
01061	SYS818	*Clonostachys candelabrum*	Ascomycota	Sordariomycetes	Hypocreales
01062	SYS794	*Eucasphaeria capensis*	Ascomycota	Sordariomycetes	Hypocreales
01063	SYS772	*Galactomyces geotrichum*	Ascomycota	Saccharomycetes	Saccharomycetales;
01064	SYS797	*Gibellulopsis nigrescens / Verticillium dahliae*	Ascomycota	Sordariomycetes	Glomerellales
01065	SYS793	*Microsphaeropsis arundinis*	Ascomycota	mitosporic Ascomycota	mitosporic Ascomycota
01066	SYS799	*Myrmecridium* sp.	Ascomycota	Sordariomycetes	mitosporic Sordariomycetes
01067	SYS773	*Paraconiothyrium sporulosum / Coniothyrium sp*	Ascomycota	Dothideomycetes	Pleosporales
01069	SYS824	*Chloridium virescens*	Ascomycota	Sordariomycetes	Chaetosphaeriales
01070	SYS845	*Chloridium virescens*	Ascomycota	Sordariomycetes	Chaetosphaeriales
01073	SYS894	*Tolypocladium inflatum*	Ascomycota	Sordariomycetes	Hypocreales
01074	SYS996	*Chloridium sp*	Ascomycota	Sordariomycetes	Chaetosphaeriales
01075	SYS994	*Paraconiothyrium sporulosum*	Ascomycota	Dothideomycetes	Pleosporales
01076		*Trichoderma* sp.	Ascomycota	Sordariomycetes	Hypocreales
01077		*Trichoderma* sp.	Ascomycota	Sordariomycetes	Hypocreales
01078		*Trichoderma* sp.	Ascomycota	Sordariomycetes	Hypocreales
01079		*Hypocrea rodmanii*	Ascomycota	Sordariomycetes	Hypocreales
01080		*Trichoderma* sp.	Ascomycota	Sordariomycetes	Hypocreales
01081		*Trichoderma* sp.	Ascomycota	Sordariomycetes	Hypocreales
01082		*Trichoderma* sp.	Ascomycota	Sordariomycetes	Hypocreales
01083		*Trichoderma viride*	Ascomycota	Sordariomycetes	Hypocreales
01084		*Trichoderma koningiopsis / Hypocrea koningii*	Ascomycota	Sordariomycetes	Hypocreales
01085		*Trichoderma* sp.	Ascomycota	Sordariomycetes	Hypocreales
01086		*Hypocrea koningii*	Ascomycota	Sordariomycetes	Hypocreales
01087		*Trichoderma harzianum* (anamorph)	Ascomycota	Sordariomycetes	Hypocreales

01088		*Trichoderma* sp.	Ascomycota	Sordariomycetes	Hypocreales
01089		*Trichoderma* sp.	Ascomycota	Sordariomycetes	Hypocreales
01090		*Trichoderma* sp.	Ascomycota	Sordariomycetes	Hypocreales
01091		*Trichoderma* sp.	Ascomycota	Sordariomycetes	Hypocreales
01092		*Trichoderma* sp.	Ascomycota	Sordariomycetes	Hypocreales
01093		*Trichoderma* sp.	Ascomycota	Sordariomycetes	Hypocreales
01094		*Trichoderma* sp.	Ascomycota	Sordariomycetes	Hypocreales
01095		*Trichoderma* sp.	Ascomycota	Sordariomycetes	Hypocreales
01096		*Trichoderma* sp.	Ascomycota	Sordariomycetes	Hypocreales
01097		*Trichoderma* sp.	Ascomycota	Sordariomycetes	Hypocreales
01098		*Hypocrea lixii*	Ascomycota	Sordariomycetes	Hypocreales
01099		*Trichoderma* sp.	Ascomycota	Sordariomycetes	Hypocreales
01100		*Trichoderma* sp.	Ascomycota	Sordariomycetes	Hypocreales
01101		*Trichoderma* sp.	Ascomycota	Sordariomycetes	Hypocreales
01102		*Trichoderma* sp.	Ascomycota	Sordariomycetes	Hypocreales
01103		*Trichoderma* sp.	Ascomycota	Sordariomycetes	Hypocreales
01104		*Trichoderma asperellum*	Ascomycota	Sordariomycetes	Hypocreales
01105		*Trichoderma* sp.	Ascomycota	Sordariomycetes	Hypocreales
01106		*Trichoderma koningii / Trichoderma fertile / Hypocrea f*[illegible]	Ascomycota	Sordariomycetes	Hypocreales
01107		*Trichoderma* sp.	Ascomycota	Sordariomycetes	Hypocreales
01108		*Trichoderma asperellum*	Ascomycota	Sordariomycetes	Hypocreales
01109		*Trichoderma* sp.	Ascomycota	Sordariomycetes	Hypocreales
01110		*Hypocrea koningii*	Ascomycota	Sordariomycetes	Hypocreales
01111		*Trichoderma* sp.	Ascomycota	Sordariomycetes	Hypocreales
01112		*Trichoderma* sp.	Ascomycota	Sordariomycetes	Hypocreales
01113		*Trichoderma harzianum* (anamorph)	Ascomycota	Sordariomycetes	Hypocreales
01115		*Trichoderma* sp.	Ascomycota	Sordariomycetes	Hypocreales
01117		*Pestalotiopsis* sp.	Ascomycota	Sordariomycetes	Xylariales
01118		*Myrothecium* sp.	Ascomycota	Sordariomycetes	Hypocreales
01119		*Beauveria* sp.	Ascomycota	Sordariomycetes	Hypocreales
01120		*Paraphaeosphaeria* sp.	Ascomycota	Dothideomycetes	Pleosporales
01121		*Metarhizium anisopliae*	Ascomycota	Sordariomycetes	Hypocreales
01122		*Coniothyrium* sp.	Ascomycota	Dothideomycetes	Pleosporales
01515	0610-002 1 A PDA	*Colletotrichum coccodes*	Ascomycota	Sordariomycetes	Glomerellales
01518	I05	*Aspergillus* sp.	Ascomycota	Eurotiomycetes	Eurotiales
01519	SYS762	*Fusarium* sp.	Ascomycota	Sordariomycetes	Hypocreales
01520	SYS673	*Penicillium* sp.	Ascomycota	Eurotiomycetes	Eurotiales
00052	C03	*Rhizoctonia solani* AG 1-1B	Basidiomycota	Agaricomycetes	Cantharellales
00056	H01	*Rhizoctonia solani* AG 2-3	Basidiomycota	Agaricomycetes	Cantharellales
00057	U01	*Rhizoctonia solani* AG 2 BI	Basidiomycota	Agaricomycetes	Cantharellales
00058	G05	*Rhizoctonia solani* AG 2-2IV	Basidiomycota	Agaricomycetes	Cantharellales
00059	J02	*Rhizoctonia solani* AG 4	Basidiomycota	Agaricomycetes	Cantharellales
00071	G10	*Rhizoctonia solani* AG 2-2-2LP	Basidiomycota	Agaricomycetes	Cantharellales
00078	P01	*Rhizoctonia solani* AG 8	Basidiomycota	Agaricomycetes	Cantharellales
00195	0629-048 1 B β WA	*Bjerkandera adusta*	Basidiomycota	Agaricomycetes	Polyporales
00979	SYS325	*Trichosporon porosum*	Basidiomycota	Tremellomycetes	Tremellales
00009	0729-005 1 A PDA	*Mucor hiemalis*	Fungi incertae sedis	-	Mucorales
00377	0629-006 1 PDA	*Absidia glauca*	Fungi incertae sedis	-	Mucorales
00406	0728-091 B PDA	*Mucor fragilis*	Fungi incertae sedis	-	Mucorales
00942	SYS321	*Cunninghamella elegans*	Fungi incertae sedis	-	Mucorales
00980	SYS480	*Umbelopsis autotrophica*	Fungi incertae sedis	-	Mucorales
00981	SYS358	*Umbelopsis isabellina*	Fungi incertae sedis	-	Mucorales
00983	SYS070	*Zygorhynchus moelleri*	Fungi incertae sedis	-	Mucorales
01049	SYS245	*Zygorhynchus moelleri*	Fungi incertae sedis	-	Mucorales
01051	SYS324	*Umbelopsis autotrophica*	Fungi incertae sedis	-	Mucorales
01052	SYS334	*Umbelopsis autotrophica*	Fungi incertae sedis	-	Mucorales
01055	SYS440	*Zygorhynchus moelleri*	Fungi incertae sedis	-	Mucorales
01056	SYS527	*Absidia glauca*	Fungi incertae sedis	-	Mucorales
01068	SYS707	*Umbelopsis isabellina*	Fungi incertae sedis	-	Mucorales
01071	SYS892	*Umbelopsis autotrophica*	Fungi incertae sedis	-	Mucorales
01072	SYS843	*Umbelopsis isabellina*	Fungi incertae sedis	-	Mucorales
01123		*Rhizopus oryzae*	Fungi incertae sedis	-	Mucorales
00955	SYS775	*Mortierella elongata*	Fungi incertae sedis	-	Mortierellales
00956	SYS899	*Mortierella gamsii*	Fungi incertae sedis	-	Mortierellales
00957	SYS356	*Mortierella horticola*	Fungi incertae sedis	-	Mortierellales
00958	SYS402	*Mortierella humilis*	Fungi incertae sedis	-	Mortierellales
00012	PAR400	*Phytophthora parasitica*	-	oomycetes	Peronosporales
00013	OP4	*Pythium aphanidermatum*	-	oomycetes	Pythiales
00018	Py PC1	*Pythium sylvaticum*	-	oomycetes	Pythiales

Maeda et al., Table S3

Table S3 Summary of the strains tested		
Order	Strains tested	Positive strains
Hypocreales	113	50
Eurotiales	23	8
Mucorales	16	1
Pleosporales	9	1
Cantharellales	7	
Sordariales	7	5
Chaetosphaeriales	4	3
Mortierellales	4	
Capnodiales	2	
Xylariales	3	
Glomerellales	2	1
Helotiales	2	
Pythiales	2	
Ophiostomatales	1	1
Peronosporales	1	
Magnaporthales	1	
Polyporales	1	
Tremellales	1	
Saccharomycetales	1	
Leotiomycetes incertae sedis	1	
mitosporic Sordariomycetes	1	
mitosporic Dothideomycetes	1	
mitosporic Ascomycota	4	
	207	70
